# Long-Tailed Multi-Label Diagnosis of Compound Faults in Wind Turbine Gearboxes via Multi-Channel Imaging of FBG Vibration Signals

**DOI:** 10.3390/s26154784

**Published:** 2026-07-28

**Authors:** Yuhan Peng, Xuetao Duan, Haoyuan Tian, Hong Liu, Tanglong Liu, Wentao Zhang, Ketan Chen, Zhiqing Shu, Weigen Chen

**Affiliations:** State Key Laboratory of Power Transmission Equipment Technology, Chongqing University, Chongqing 400044, China

**Keywords:** wind turbine gearbox, fiber Bragg grating, compound fault diagnosis, multi-label classification, long-tailed distribution

## Abstract

Wind power plays an important role in renewable energy generation, and the reliability of wind turbine gearboxes directly affects turbine operation and maintenance. Compound gear fault diagnosis remains challenging because multiple fault components may coexist and compound fault samples are often limited, leading to long-tailed data distributions. To address this problem, this study proposes a long-tailed multi-label diagnostic framework based on fiber Bragg grating (FBG) acceleration signals and multi-channel time-series imaging. Missing tooth, pitting, and tooth breakage faults are encoded as three independent labels to represent healthy, single-fault, double compound fault, and triple compound fault conditions. The one-dimensional FBG wavelength-shift signals are transformed into GASF-GADF-MTF three-channel images, which describe amplitude angular correlation, dynamic angular difference, and state transition information. A ResNet18-SE network trained with Focal Loss is developed to improve the recognition of minority compound fault samples. Experimental results show that the proposed method achieves an Exact Match Accuracy of 0.9950 and a Macro-F1 of 0.9980 on the Balanced dataset. Under the severe LT50 setting, it achieves an Exact Match Accuracy of 0.9739 and an F1_123_ of 0.9469. These results demonstrate the effectiveness of the proposed framework for FBG-based long-tailed compound fault diagnosis.

## 1. Introduction

The drivetrain of wind turbines is susceptible to performance degradation under long-term alternating loads, stochastic wind speed disturbances, and complex operating conditions. As a core transmission component, the gearbox health status directly affects the reliability and maintenance cost of the whole turbine. Typical gear faults, such as tooth surface pitting, missing teeth, tooth breakage, and wear, may lead to unplanned downtime or drivetrain failure if they are not identified in time. Therefore, reliable condition monitoring and intelligent fault diagnosis of wind turbine gearboxes are of great engineering significance. To address this issue, extensive studies have been conducted on signal processing, feature extraction, and intelligent classification models [1,2,3]. Among them, vibration-response-based monitoring remains one of the most mature and widely used technical approaches [4].

In current engineering applications, vibration signals are commonly acquired using conventional electrical accelerometers, such as piezoelectric or MEMS accelerometers, which convert mechanical acceleration or vibration into electrical output signals. However, in wind farm environments, strong electromagnetic interference, transient disturbances, restricted nacelle space, and long-distance cabling may increase the difficulty of stable vibration monitoring and distributed sensor deployment [5]. In contrast, fiber Bragg grating (FBG) sensors have attracted increasing attention in rotating machinery condition monitoring because of their electromagnetic immunity, multiplexing capability, compact size, and suitability for long-distance transmission [6,7,8]. It should be noted that FBG vibration measurements are obtained by demodulating the dynamic shift in the Bragg wavelength induced by strain. This signal generation mechanism differs from that of conventional electrical accelerometers, resulting in different noise characteristics and statistical properties. Therefore, constructing discriminative feature representations and learning frameworks suitable for FBG vibration signals is important for reliable optical-fiber-based intelligent monitoring.

In data-driven fault diagnosis, time-series imaging provides an effective way to exploit the feature extraction ability of convolutional neural networks (CNNs). Existing studies have attempted to transform one-dimensional vibration signals into two-dimensional image representations. For example, Gramian Angular Summation Field (GASF), Gramian Angular Difference Field (GADF), and Markov Transition Field (MTF) encode time-series signals from different perspectives, including amplitude angular correlation, dynamic angular difference, and state transition probability [9,10,11,12]. For compound gear fault diagnosis, vibration responses often contain the superposition of multiple excitation sources and coupled fault components. A single representation may be insufficient to fully characterize such complex patterns. Therefore, integrating GASF, GADF, and MTF into a multi-channel image representation is expected to improve the discriminative feature representation of FBG vibration signals.

In addition to signal representation, compound fault diagnosis in wind turbine gearboxes also faces two important learning challenges. First, fault modes in practical operation are not always mutually exclusive. Multiple fault components may occur simultaneously at different transmission stages. If a traditional single-label classification strategy is adopted, each compound fault combination must be defined as an independent class. This strategy not only increases the number of predefined categories but also fails to directly indicate the presence or absence of each basic fault component. Multi-label classification provides a more suitable formulation for this problem, because each output label corresponds to one basic fault component [13].

Second, compound fault samples are usually more difficult to obtain than normal or single-fault samples, leading to a long-tailed data distribution. Under such conditions, head classes with abundant samples tend to dominate model training, while tail compound fault classes are insufficiently learned [14]. To mitigate this problem, class re-weighting, hard sample mining, and Focal Loss have been introduced to adjust the effective contribution of different samples during training [15,16]. However, long-tailed multi-label diagnosis of compound faults based on FBG vibration signals remains challenging, because the model must simultaneously learn different fault components and distinguish their overlapping combinations. A systematic framework and ablation analysis are therefore required to evaluate the influence of feature representation, loss function, attention mechanism, and backbone network on tail compound fault recognition.

Based on the above analysis, this paper proposes a long-tailed multi-label diagnostic framework for compound gear faults in wind turbine gearboxes using FBG vibration signals and multi-channel time-series imaging. The main contributions of this study are summarized as follows:

(1) A multi-label diagnostic framework for compound gear faults in wind turbine gearboxes is constructed based on FBG vibration signals. Missing tooth, pitting, and tooth breakage are encoded as three independent fault labels, enabling the direct identification of basic fault components in single-fault and compound-fault conditions.
(2) A GASF-GADF-MTF multi-channel time-series imaging strategy is proposed for FBG wavelength-shift signals. By integrating amplitude angular correlation, dynamic angular difference, and state transition probability information, the proposed representation enhances the characterization of compound gear fault features.
(3) The long-tailed multi-label diagnostic performance of compound gear faults is systematically analyzed. Balanced and long-tailed datasets are constructed, and the effects of different loss functions, attention mechanisms, and representative backbone networks on tail compound fault recognition are compared.

## 2. Materials and Methods

### 2.1. Experimental Setup and Multi-Label Fault Configuration

**Figure 1:**
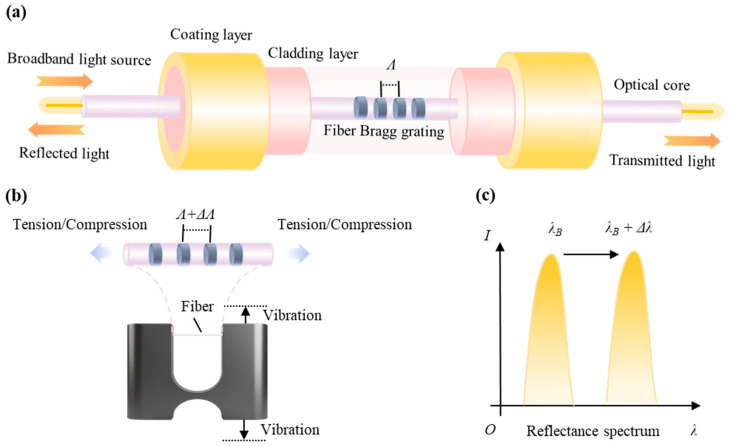
Schematic diagram of the FBG sensing principle and vibration-induced center wavelength shift: (**a**) propagation of the broadband light source, reflected light, and transmitted light in the FBG; (**b**) tensile/compressive strain of the fiber induced by vibration; (**c**) shift in the FBG reflectance spectrum.

An FBG is a wavelength-selective sensing element formed by a periodic modulation of the refractive index in the fiber core. As shown in Figure 1a, the dark periodic regions in the fiber core represent the periodic refractive-index modulation planes in the fiber Bragg grating, and the distance between two adjacent refractive-index modulation planes is the grating period Λ. When broadband light propagates along the optical fiber and reaches the grating region, the wavelength component satisfying the Bragg reflection condition is reflected, while the remaining components continue to be transmitted. The transmitted light in Figure 1a refers to the optical signal that is not reflected by the FBG; its power may be affected by fiber length, connector loss, bending loss, and fiber attenuation. However, the measurement output of the FBG accelerometer is the Bragg wavelength shift rather than the absolute optical intensity. A narrow reflection peak is therefore formed in the reflected spectrum, and its central wavelength is defined as the Bragg wavelength, which can be expressed as

(1) $$\lambda_{B}=2n_{\mathrm{eff}}\Lambda$$

where *λ*_B_ is the Bragg wavelength, *n*_eff_ is the effective refractive index of the fiber core, and *λ* is the grating period. This relationship indicates that the Bragg wavelength is jointly determined by the effective refractive index and the grating period. When axial tensile or compressive strain is applied to the grating region, the grating period changes with fiber deformation, while the effective refractive index is also modified by the photoelastic effect. Consequently, the Bragg wavelength shifts accordingly. Under the linear small-deformation assumption, the relative wavelength shift can be approximately related to the axial strain as

(2) $$\frac{\Delta \lambda_{B}}{\lambda_{B}}=(1-p_{e})\varepsilon$$

where Δ*λ*_B_ denotes the Bragg wavelength shift, *p*_e_ is the effective photoelastic coefficient of the fiber, and *ε* is the axial strain of the fiber. This relationship shows that the axial strain applied to the FBG can be characterized by monitoring the Bragg wavelength shift.

For an FBG accelerometer, external vibration needs to be converted into axial strain in the optical fiber through an elastic sensing structure. As shown in Figure 1b, when gearbox vibration is transmitted to the sensor base, the inertial mass undergoes slight relative motion with respect to the base under inertial force, causing elastic deformation near the flexure hinge. Since the FBG spans the flexure hinge region and is fixed at both ends, this structural deformation produces periodic tensile or compressive strain in the grating, resulting in a time-varying shift in the Bragg wavelength. Figure 1c further illustrates the shift in the FBG reflection spectrum induced by axial strain. Therefore, gearbox vibration can be converted by the hinge-type sensing structure into a dynamic Bragg wavelength variation, providing the signal basis for subsequent compound gear fault feature extraction and multi-label diagnosis.

**Figure 2:**
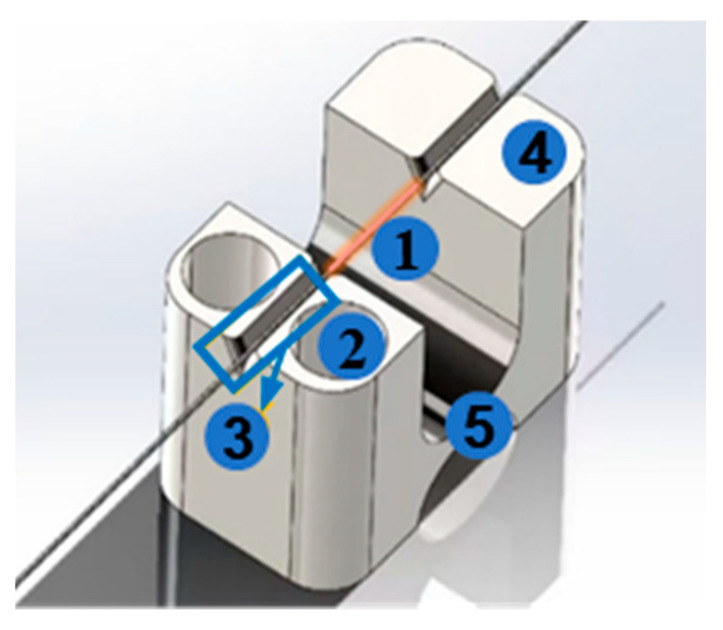
Structural schematic of the FBG accelerometer: 1—FBG sensing segment; 2—counterbored holes; 3—V-groove; 4—proof mass; 5—circular-notch flexure hinge.

**Figure 3:**
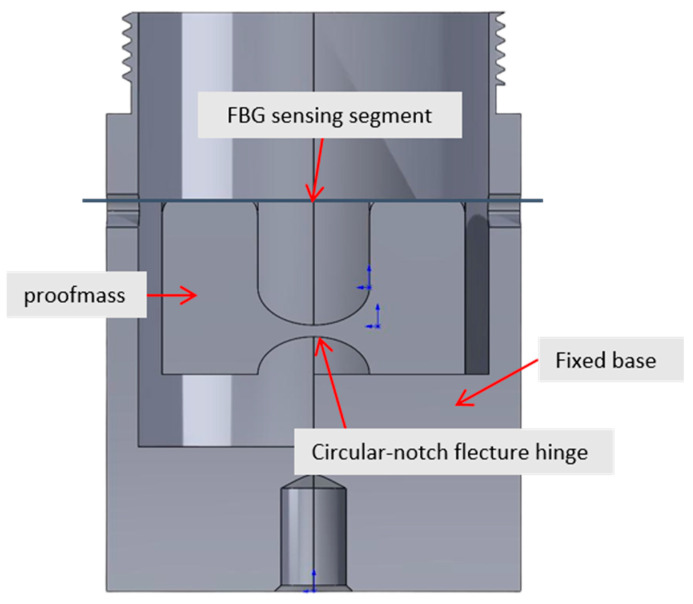
Two-dimensional schematic of the hinge-type FBG vibration sensor, showing the FBG sensing segment, proof mass, fixed base, and circular-notch flexure hinge.

As shown in Figure 2, the hinge-type FBG vibration sensor used in this study mainly consists of an FBG sensing element, counterbored holes, a V-groove, a proof mass, and a circular-notch flexure hinge. To further clarify the internal mechanical structure of the sensor, a two-dimensional schematic view is additionally provided in Figure 3. As shown in Figure 3, the proof mass corresponds to the suspended block on one side of the flexure hinge, while the fixed base corresponds to the supporting part rigidly connected to the sensor body. The optical fiber passes through the sensor structure, and the FBG sensing segment is arranged in the deformation-sensitive region. The sensor can be regarded as an equivalent spring–mass system, in which the proof mass acts as the equivalent mass and the circular-notch flexure hinge provides the dominant equivalent stiffness. When external vibration is applied to the sensor, the proof mass generates inertial force, causing elastic deformation of the flexure hinge and inducing axial strain variation in the FBG sensing segment through the bonded structure. This strain variation leads to a Bragg wavelength shift, and vibration information can therefore be obtained by demodulating the wavelength shift.

**Figure 4:**
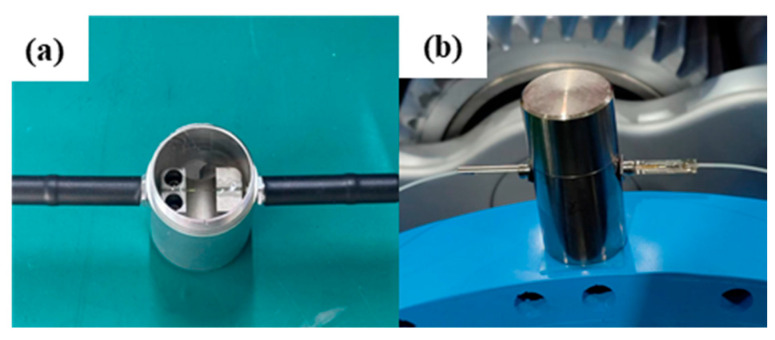
Structure of the hinge-type FBG accelerometer: (**a**) internal structure of the opened sensor; (**b**) packaged sensor.

Based on the sensing principle of the hinge-type FBG vibration sensor described above, the hinge-type FBG sensor used in this study is shown in Figure 4. Figure 4a presents the internal structure of the opened sensor. The sensor adopts a monolithic metallic structure, with the left section serving as the fixed base. Two mounting holes are arranged on the base to fix the monolithic metallic block onto the internal base of the sensor package. The right section functions as the inertial mass, which can generate slight vertical oscillation relative to the fixed base under vibration input from the packaged base. The FBG is arranged along the axial direction of the sensor, passes through the central V-groove, and is fixed at both ends of the monolithic metallic block using epoxy adhesive. This configuration enables the structural deformation near the flexure hinge to be effectively transferred to the FBG sensing region. To protect the internal optical fiber and adhesive fixation points and to improve the structural stability of the sensor during installation and vibration testing, the sensing module was packaged. The FBG accelerometer was tested using a standard vibration calibration system. During calibration, the sensor was subjected to a constant acceleration excitation of 0.5 g, and the corresponding Bragg wavelength shift was recorded. The sensitivity unit pm/g denotes the Bragg wavelength shift induced by one unit of gravitational acceleration, where pm represents the picometer-level wavelength shift and g represents the gravitational acceleration unit, with 1 g = 9.81 m/s^2^. The packaged sensor is shown in Figure 4b. The main parameters of the hinge-type FBG accelerometer are shown in Table 1.

**Figure 5:**
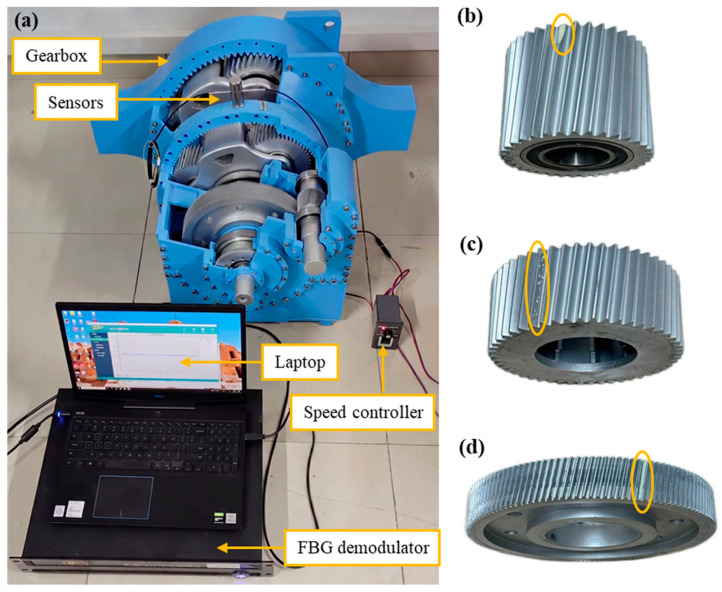
Gearbox vibration signal acquisition platform and gear fault configurations: (**a**) experimental setup for FBG vibration signal acquisition; (**b**) tooth breakage fault of the second-stage planetary gear; (**c**) pitting fault of the first-stage planetary gear; (**d**) missing tooth fault of the high-speed shaft gear.

The gearbox vibration signal acquisition platform is shown in Figure 5a. During the experiment, the input speed of the gearbox was maintained at 100 rpm by adjusting the speed controller knob. The hinge-type FBG vibration sensor was fixed on the gearbox housing corresponding to the position above the first-stage planetary gear to acquire vibration responses during gearbox operation. The acquired FBG center wavelength variation signal was transmitted through the optical fiber to an FBG interrogator (YOFC-FBG-8-100, Yangtze Optical Fibre and Cable Joint Stock Limited Company, Wuhan, China), and the real-time wavelength data were displayed and recorded using a laptop. The direct output recorded by the FBG interrogator is the Bragg center-wavelength variation induced by structural strain. Since the hinge-type FBG sensor was used as an acceleration sensing element and calibrated with acceleration sensitivity, the demodulated wavelength-shift response can represent the vibration-induced acceleration response of the gearbox. Therefore, although the direct output recorded by the interrogator is the FBG center wavelength variation, the demodulated vibration response is referred to as the FBG acceleration signal after sensor calibration. The main parameters of the FBG interrogator are listed in Table 2. To construct the compound gear fault diagnosis dataset, the high-speed shaft gear, first-stage planetary gear, and second-stage planetary gear were each configured under normal or faulty conditions. Specifically, the fault of the second-stage planetary gear was set as tooth breakage, as shown in Figure 5b; the fault of the first-stage planetary gear was set as pitting, as shown in Figure 5c; and the fault of the high-speed shaft gear was set as a missing tooth, as shown in Figure 5d. The faulty gears used in this study were directly fabricated by 3D printing according to predefined defect geometry models, rather than being generated by random wear during operation. Therefore, the locations and morphologies of the missing-tooth, pitting, and tooth-breakage defects were determined before the experiment, ensuring the controllability and repeatability of different fault conditions. For the missing-tooth fault, even if the damaged region is located only at the tooth tip or a local part of the tooth surface, it interrupts the continuous meshing relationship between the damaged tooth and the mating gear. When the damaged tooth enters the meshing region, the actual contact length and contact area are reduced, the local meshing stiffness decreases, and the load is redistributed among adjacent teeth. When it leaves the meshing region, the meshing stiffness changes abruptly and transmission-error fluctuation is introduced. This periodic variation in contact stiffness excites impact and modulation components, which are transmitted through the gearbox housing to the FBG sensor. Therefore, a local missing-tooth defect does not produce a vibration response identical to that of a normal gear, but appears in the FBG signal as periodic disturbances and texture changes. Based on different combinations of these three basic gear faults, eight operating conditions were constructed, as listed in Table 3, and the corresponding FBG vibration response data were collected. Each operating condition was further encoded using a three-bit fault vector to indicate the presence or absence of the corresponding basic fault components.

### 2.2. GASF-GADF-MTF Time Series Image Representation

**Figure 6:**
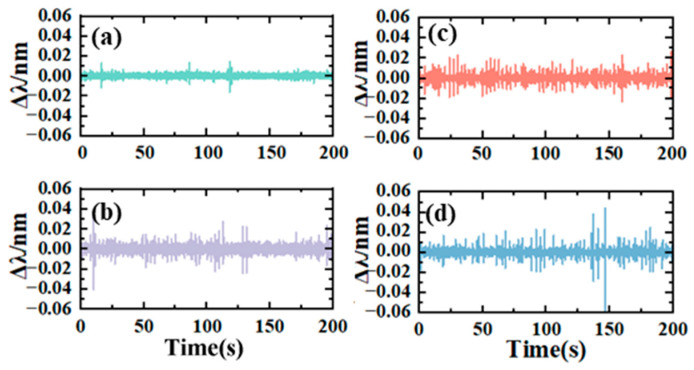
Representative FBG acceleration time-series signals under different gear conditions: (**a**) Class 0: healthy state; (**b**) Class 1: high-speed shaft missing tooth; (**c**) Class 12: compound fault of missing tooth and first-stage planetary gear pitting; (**d**) Class 123: triple compound fault of missing tooth, pitting, and partial tooth breakage.

The FBG vibration response acquired from the gearbox is a one-dimensional time series. Figure 6 shows representative FBG acceleration time-series signals under different gear conditions. Compared to the healthy state, the fault conditions show stronger amplitude fluctuations, more obvious impact components, and increased non-stationary characteristics. These time-series samples provide an intuitive illustration of the FBG acceleration responses before multi-channel time-series image encoding. To exploit the ability of convolutional neural networks to extract texture features from two-dimensional representations, the FBG signal was converted into a three-channel time series image based on GASF, GADF, and MTF [9,10,11,12]. Specifically, the one-dimensional FBG center-wavelength variation signal was processed through envelope extraction, segment-wise RMS dimensionality reduction, normalization, and image encoding. GASF, GADF, and MTF were generated from the same reduced sequence and assigned to the R, G, and B channels, respectively, to construct a pseudo-color image as the input of the ResNet18-SE model. The procedure includes envelope extraction, segment-wise dimensionality reduction, normalization, two-dimensional image encoding, and channel fusion.

For an FBG signal sample, the sequence can be expressed as

(3) $$X=x_{1},x_{2},\ldots ,x_{L}$$

where *L* is the sample length, and *x_i_* denotes the FBG center wavelength variation at the *i*-th sampling point. The direct current component was first removed from the signal, and the Hilbert transform was then used to extract the envelope:

(4) $$e_{i}=\left|H\left(x_{i}-\bar{x}\right)\right|$$

where $\bar{x}$ is the mean value of the signal sample, H(⋅) denotes the Hilbert transform, and *e_i_* is the extracted envelope sequence. The envelope sequence was then divided into *N* consecutive segments, and the root mean square value of each segment was calculated to obtain the reduced envelope feature sequence:

(5) $$S=s_{1},s_{2},\ldots ,s_{N}$$

(6) $$s_{j}=\sqrt{\frac{1}{\left|\Omega_{j}\right|}\sum_{i\in \Omega_{j}}e_{i}^{2}},\;\;\;\;j=1,2,\ldots ,N$$

where $\Omega_{j}$ denotes the set of sampling points in the *j*-th segment, and $\left|\Omega_{j}\right|$ is the number of sampling points in that segment. In this study, *N* was set to 224 to generate encoded images with a size of 224×224. The sampling frequency affects the retained frequency content of the original FBG vibration signal and the number of sampling points in each signal window. These factors further affect the envelope extraction, segment-wise RMS dimensionality reduction, and the texture details of the final encoded images. In this study, all samples were generated using the same sampling frequency and image construction procedure. RMS values are used in this study during segment-wise dimensionality reduction to construct the reduced envelope feature sequence for image encoding. However, this study does not classify gear conditions using acceleration RMS alone. Amplitude-based indicators can describe the overall vibration level, but they may be insufficient to distinguish similar or compound fault patterns. Therefore, the proposed framework uses GASF-GADF-MTF encoded images and the ResNet18-SE model for multi-label fault diagnosis.

To unify the amplitude scale of different samples and reduce the influence of local abnormal peaks on the normalization range, a percentile normalization method based on the training set was adopted. Specifically, the 5th and 95th percentiles of all reduced sequences in the training set were calculated and denoted as $s_{\min,\mathrm{tr}}$ and $s_{\max,\mathrm{tr}}$, respectively. The same normalization parameters were then applied to the training, validation, and test sets:

(7) $${\hat{s}}_{j}=2\frac{s_{j}-s_{\min,\mathrm{tr}}}{s_{\max,\mathrm{tr}}-s_{\min,\mathrm{tr}}}-1$$

After normalization, values outside the interval [−1, 1] were clipped to the boundary values, yielding the normalized sequence

(8) $$\tilde{S}={\tilde{s}}_{1},{\tilde{s}}_{2},\ldots ,{\tilde{s}}_{N}$$

where ${\hat{s}}_{j}$ is the percentile-normalized feature value, and ${\tilde{s}}_{j}$ is the clipped normalized feature value. Using the 5th and 95th percentiles estimated from the training set as the normalization bounds reduces the effect of a small number of extreme amplitudes on the overall scale, while preventing information from the validation and test sets from being involved in the estimation of normalization parameters.

The normalized sequence $\tilde{S}$ was then mapped into the angular domain:

(9) $$\theta_{j}=\arccos({\tilde{s}}_{j}),\;\;\;\;j=1,2,\ldots ,N$$

GASF constructs a two-dimensional matrix by calculating the cosine of the angular summation between different temporal positions. It is used to characterize amplitude angular correlations in the sequence:

(10) $$G_{S}(i,j)=\cos(\theta_{i}+\theta_{j})$$

where $G_{S}(i,j)$ is the element in the *i*-th row and *j*-th column of the GASF matrix. GASF maps the amplitude variation and global correlation structure of a one-dimensional sequence into two-dimensional texture features, thereby reflecting the overall pattern changes caused by gear meshing vibration and impacts associated with gear faults.

GADF constructs a two-dimensional matrix by calculating the sine of the angular difference between different temporal positions. It is used to characterize dynamic variation relationships. According to the implementation in this study, GADF is defined as

(11) $$G_{D}(i,j)=\sin(\theta_{j}-\theta_{i})$$

where $G_{D}(i,j)$ is the element in the *i*-th row and *j*-th column of the GADF matrix. Compared to GASF, GADF emphasizes the relative variation between different temporal positions, which helps characterize local differences caused by fault impacts and nonstationary fluctuations.

MTF describes the dynamic evolution of the FBG vibration signal from the perspective of state transition. The normalized sequence $\tilde{S}$ was divided into *Q* equal-width state intervals within [−1, 1], and each temporal position was assigned to a corresponding state. According to the state transitions between adjacent temporal positions, a first-order state transition probability matrix was obtained:

(12) $$P_{mn}=\frac{C_{mn}}{\sum_{n=1}^{Q}C_{mn}}$$

where $C_{mn}$ denotes the number of transitions from state *m* to state *n*, and $P_{mn}$ represents the corresponding transition probability. The transition probability matrix was then expanded into an MTF matrix according to the states corresponding to any two temporal positions:

(13) $$M\left(i,j\right)=P_{qi,qj}$$

where *q_i_* and *q_j_* are the states corresponding to the *i*-th and *j*-th temporal positions, respectively. MTF represents the transition relationships among different amplitude states in the FBG vibration signal and is used to describe state changes caused by fault impacts and nonstationary vibration.

GASF, GADF, and MTF characterize the same FBG vibration signal from three perspectives: amplitude angular correlation, dynamic angular difference, and state transition probability. In this study, GASF, GADF, and MTF were used as three input channels and fused along the channel dimension:

(14) $$I=\mathrm{Stack}(G_{S},G_{D},M)$$

where *I* denotes the constructed GASF-GADF-MTF three-channel time series image. The size of the final input image is expressed as

(15) $$I\in {\mathbb{R}}^{224\times 224\times 3}$$

The constructed image was subsequently used as the input of the multi-label diagnostic model.

**Figure 7:**
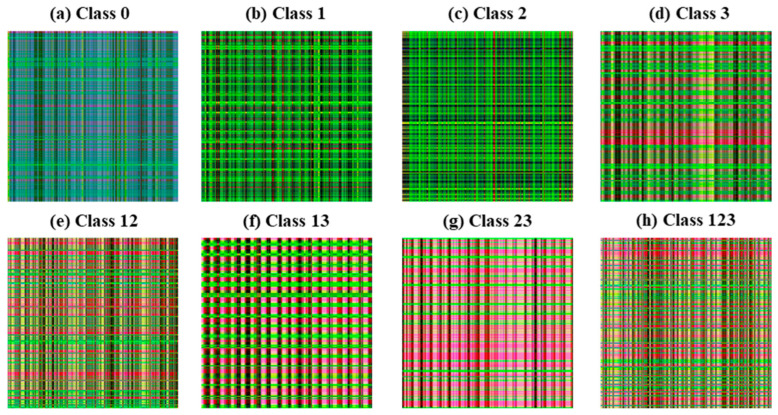
Representative GASF-GADF-MTF encoded images under different gearbox operating conditions: (**a**) 0-healthy; (**b**) 1-high-speed shaft gear missing tooth; (**c**) 2-first-stage planetary gear pitting; (**d**) 3-second-stage planetary gear tooth breakage; (**e**) 12-missing tooth + pitting; (**f**) 13-missing tooth + tooth breakage; (**g**) 23-pitting + tooth breakage; (**h**) 123-missing tooth + pitting + tooth breakage.

Figure 7 shows representative encoded images generated from FBG vibration signals under different operating conditions. In the normal state, the encoded image presents relatively regular and uniform texture distributions. In contrast, defective gears introduce impact, modulation, and non-stationary components into the FBG vibration response, resulting in more pronounced local texture variations and non-uniform color distributions in the encoded images. The texture patterns vary among normal, single-fault, and compound-fault conditions, indicating that the GASF-GADF-MTF representation can transform one-dimensional FBG vibration responses into discriminative two-dimensional image patterns for subsequent feature learning. It should be noted that the GASF-GADF-MTF encoded images are not frequency spectra; therefore, gear meshing frequencies cannot be directly observed as spectral peaks in these images. Instead, meshing-related periodic components are indirectly represented through texture patterns related to amplitude angular correlation, dynamic angular difference, and state transition behavior. It should be noted that these encoded images are used as feature representations for network learning rather than as a manual diagnostic criterion. Missing tooth and tooth breakage may be difficult to distinguish only by visually observing vibration waveforms or encoded images. In this study, their distinction is achieved by the proposed GASF-GADF-MTF representation and the ResNet18-SE multi-label diagnostic model, where GASF, GADF, and MTF describe amplitude angular correlation, dynamic angular difference, and state transition information, respectively. For compound faults such as Figure 7h, missing tooth, pitting, and tooth breakage are not manually separated from the encoded image. Instead, they are represented by three independent fault labels, and the ResNet18-SE model outputs the predicted probabilities of the three labels to identify the coexistence of the basic fault components. This study focuses on fault-category recognition rather than the quantitative identification of defect size or severity.

### 2.3. Dataset Construction and Long-Tailed Distribution Design

To avoid data leakage caused by adjacent samples being distributed across different subsets, the FBG vibration signals of each operating condition were first divided into training, validation, and test sets in chronological order, with proportions of 0.70, 0.15, and 0.15, respectively. The FBG vibration signals in each subset were then subjected to dimensionality reduction and image encoding according to the method described in Section 2.2. Signal samples crossing the boundaries between the training, validation, and test sets were excluded. After image encoding, a quality screening procedure was performed to remove invalid images with abnormal pixel distributions. Finally, the number of images in each class was aligned with that of the class containing the fewest images, ensuring that all classes in the balanced dataset had exactly the same number of image samples. Specifically, after image encoding and quality screening, the class containing the fewest valid images was used as the reference, and the other classes were downsampled to the same number of images.

To investigate the influence of class imbalance on compound gear fault diagnosis, three long-tailed training sets, namely LT10, LT20, and LT50, were further constructed based on the Balanced dataset. The long-tailed imbalance ratio is defined as

(16) $$\rho =\frac{N_{\max}}{N_{\min}}$$

where *N*_max_ and *N*_min_ denote the numbers of samples in the head class and the tail class in the training set, respectively. Accordingly, LT10, LT20, and LT50 correspond to head-to-tail sample ratios of 10, 20, and 50, respectively. The long-tailed datasets were constructed only by class-wise downsampling of the training set, while the validation and test sets were kept identical to those of the balanced dataset to ensure a fair comparison under different training distributions.

Considering that normal operating samples are generally easier to obtain in practical condition monitoring, whereas compound fault samples are relatively scarce, a hierarchical long-tailed training distribution was designed according to fault complexity. The long-tailed datasets were constructed to simulate practical diagnostic scenarios in which normal and single-fault samples are relatively easier to obtain, whereas compound faults, especially triple compound faults, are much rarer. For example, in LT50, the head class contains 4050 training samples, while the triple compound fault class contains 81 training samples, giving an imbalance ratio of 50. The normal condition 0 had the largest number of training samples; the single-fault classes 1, 2, and 3 had the same secondary sample size; the double compound fault classes 12, 13, and 23 had the same lower sample size; and the triple compound fault class 123 had the fewest training samples. The training sample distributions of the balanced, LT10, LT20, and LT50 datasets are listed in Table 4.

**Figure 8:**
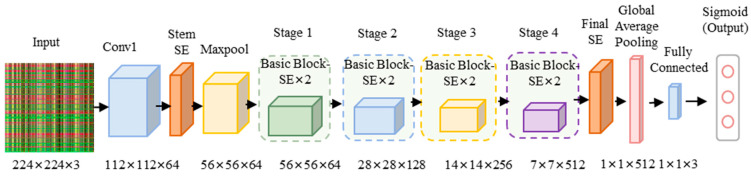
Architecture of the ResNet18-SE multi-label diagnostic model using GASF-GADF-MTF time-series images.

To effectively address the multi-label classification task for compound fault diagnosis in gearboxes with long-tailed data distributions, we propose an attention-enhanced network based on the standard ResNet18 architecture. The overall framework of the proposed model is illustrated in Figure 8. Detailed spatial dimensions and channel variations in the feature maps during forward propagation are explicitly annotated in the figure.

While retaining the hierarchical feature extraction backbone of the original ResNet, three core modifications are introduced to our model to adapt to the complex and non-mutually exclusive feature representations inherent in multi-label scenarios:

1. Early Feature Calibration (Stem Attention): A global Squeeze-and-Excitation (SE) attention module (Stem SE-Attention) is seamlessly inserted immediately after the initial convolutional layer. This design aims to suppress background noise at the earliest stage and emphasize critical channel responses from the input GASF-GADF-MTF pseudo-color images.
2. Attention-Embedded Residual Blocks (Basic Block-SE): The SE attention mechanism is embedded into each basic residual block across all four stages. This block-level integration enables dynamic feature recalibration, allowing the network to continuously focus on highly informative features during deep forward propagation.
3. Global Refinement and Multi-label Classification Head (Final Attention and Sigmoid): Prior to the Global Average Pooling (GAP) layer, a Final SE-Attention module is applied to perform an ultimate global refinement on the high-level semantic features. Furthermore, after the fully connected layer maps the aggregated features into a 3-dimensional vector, a Sigmoid activation function is employed to output the independent predicted probabilities for the three target labels, perfectly aligning with the multi-label nature of the task.

### 2.4. Squeeze-and-Excitation Attention Mechanism

In compound fault diagnosis, different types of faults exhibit varying sensitivities across different feature channels. To enable the network to adaptively focus on the most discriminative features and suppress irrelevant background noise, the Squeeze-and-Excitation (SE) attention mechanism [17] is systematically integrated into the proposed architecture. The SE module consists of three sequential operations: squeeze, excitation, and reweighting.

First, the squeeze operation aggregates the global spatial information of the input feature map $X\in {\mathbb{R}}^{C\times H\times W}$ into a channel descriptor $z\in {\mathbb{R}}^{C\times 1\times 1}$. This is achieved by applying Global Average Pooling (GAP) across the spatial dimensions H×W:

(17) $$z_{c}=\frac{1}{H\times W}\sum_{i=1}^{H}\sum_{j=1}^{W}x_{c}(i,j)$$

where $x_{c}\left(i,j\right)$ represents the value at position $\left(i,j\right)$ of the *c*-th channel.

Following the squeeze operation, the excitation mechanism is employed to capture channel-wise dependencies. This is implemented via a bottleneck architecture composed of two fully connected (FC) layers. The mechanism is formulated as

(18) $$s=\sigma (W_{2}\delta (W_{1}z))$$

where δ denotes the ReLU activation function, and σ refers to the Sigmoid function. $W_{1}\in {\mathbb{R}}^{\frac{C}{r}\times C}$ serves as a dimensionality reduction layer with a reduction ratio *r* (empirically set to 16 in our model to balance complexity and performance), while $W_{2}\in {\mathbb{R}}^{C\times \frac{C}{r}}$ acts as a dimensionality increasing layer.

Finally, in the reweighting phase, the generated channel weights *s* are multiplied channel-by-channel with the original input feature map *X* to obtain the recalibrated features $\tilde{X}$:

(19) $${\tilde{x}}_{c}=s_{c}\cdot x_{c}$$

Through this mechanism, the network dynamically amplifies critical fault signatures and attenuates redundant information.

### 2.5. Objective Function for Long-Tailed Multi-Label Classification

The compound fault diagnosis of gearboxes essentially constitutes a multi-label classification problem. To adapt to the non-mutually exclusive characteristics of the target labels, the conventional categorical cross-entropy loss is inadequate. Instead, the multi-label loss is typically formulated as the sum of independent Binary Cross-Entropy (BCE) losses for each class.

However, in realistic industrial scenarios, mechanical fault data naturally exhibits a long-tailed distribution. Training solely with the standard BCE loss leads to gradient domination by the easily classifiable head-class samples, severely degrading the model’s sensitivity to tail-class compound faults.

To effectively mitigate the impact of the long-tailed distribution and encourage the network to focus on hard-to-classify samples, this paper adopts the Focal Loss [16] as the core objective function. Specifically, let y∈{0,1} be the ground truth for a certain label and p∈[0,1] be the network’s predicted probability for that label after the Sigmoid activation. We first define *pt* as the estimated probability associated with the actual ground truth:

(20) $$p_{t}=\left\{\begin{array}{ll} p, & \mathrm{if}\;y=1\\ 1-p, & \mathrm{if}\;y=0 \end{array}\right.$$

By incorporating a modulating factor ${\left(1-p_{t}\right)}^{\gamma }$ into the standard BCE, the proposed Multi-label Focal Loss is mathematically defined as

(21) $$L_{Focal}=-{\left(1-p_{t}\right)}^{\gamma }\log(p_{t})$$

where *γ* ≥ 0 is a tunable focusing parameter that smoothly adjusts the rate at which easy examples are down-weighted.

In our proposed framework, based on hyperparameter tuning, we set *γ* = 2.0. Through this dynamic weighting mechanism, the loss contributed by easily classified majority samples (where $p_{t}\to 1$) is theoretically heavily suppressed. This design is fundamentally intended to break the gradient dominance of the head classes and dynamically steer the optimization process towards the difficult, minority compound fault samples, thereby providing a robust mathematical foundation for handling the extreme data imbalance in realistic diagnostic scenarios.

### 2.6. Experimental Settings and Evaluation Metrics

To evaluate the effectiveness of the proposed method, all experimental validations were conducted on a deep learning workstation. The hardware platform was equipped with an AMD Ryzen 7 260H processor, 16 GB of RAM, and an NVIDIA GeForce RTX 5060 Laptop GPU. The software environment was built upon the PyTorch 2.10 framework with CUDA acceleration to ensure efficient parallel computation.

During the data preprocessing phase, all raw fault images were uniformly resized to a spatial resolution of 224 × 224 pixels to fit the input dimensions of the network.

In the network training phase, the Adam optimizer [18] was employed to dynamically update the network weights, with a weight decay of 1 × 10^−4^ to prevent structural overfitting. The initial learning rate was empirically set to 3 × 10^−4^. To ensure robust convergence, the mini-batch size was set to 64. The entire optimization process was executed over a maximum of 100 epochs.

Furthermore, to prevent the model from plateauing in sub-optimal local minima, a dynamic learning rate scheduling strategy was incorporated. Specifically, if the validation loss failed to improve for 5 consecutive epochs, which served as the patience threshold, the learning rate was automatically reduced by a factor of 0.5. The best-performing model weights on the validation set were explicitly preserved for subsequent performance evaluation and comparative analysis.

## 3. Results

### 3.1. Evaluation Metrics

Evaluating a multi-label fault diagnosis model requires comprehensive metrics to assess instance-level correctness and label-level predictive ability. To evaluate the proposed model under long-tailed distributions and complex overlapping faults, five metrics are adopted: Exact Match Accuracy, Hamming Loss, Macro-F1, Composite F1 Mean, and *F*1_123_.

Let *N* denote the total number of samples and *L* the total number of fault labels. Let $y_{i}\in {\left\{0,1\right\}}^{L}$ and ${\hat{y}}_{i}\in {\left\{0,1\right\}}^{L}$ represent the ground-truth label vector and the predicted label vector for the *i*-th sample, respectively.

1. Exact Match Accuracy

Exact Match Accuracy is the most stringent metric in multi-label classification. A prediction is considered correct only if all predicted labels for a sample perfectly match the ground truth, reflecting reliability in practical industrial scenarios:

(22) $$\mathrm{EMA}=\frac{1}{N}\sum_{i=1}^{N}I(y_{i}={\hat{y}}_{i})$$

where I is the indicator function returning 1 if the condition is true and 0 otherwise.

2. Hamming Loss

Hamming Loss evaluates the fraction of incorrectly predicted labels to the total number of labels. It penalizes individual label prediction errors rather than the entire sample, providing a granular view of model stability:

(23) $$\mathrm{HL}=\frac{1}{N\times L}\sum_{i=1}^{N}\sum_{j=1}^{L}\left(y_{i,j}⊕{\hat{y}}_{i,j}\right)$$

where ⊕ denotes the logical XOR operation.

3. Macro-F1

The F1-score is the harmonic mean of Precision and Recall. To address the inherent long-tailed distribution of the industrial dataset, the Macro-F1 score is employed. It calculates the metric independently for each label and takes the unweighted average, treating minority classes with the same importance as majority classes:

(24) $$\begin{array}{c} P_{j}=\frac{TP_{j}}{TP_{j}+FP_{j}}\\ R_{j}=\frac{TP_{j}}{TP_{j}+FN_{j}}\\ \text{Macro-F1}=\frac{1}{L}\sum_{j=1}^{L}\frac{2\times P_{j}\times R_{j}}{P_{j}+R_{j}} \end{array}$$

where $TP_{j}$, $FP_{j}$, and $FN_{j}$ are true positives, false positives, and false negatives for the *j*-th fault label, respectively.

4. Composite F1 Mean and $F1_{123}$

To evaluate the model’s ability to disentangle overlapping features when multiple faults occur simultaneously, we introduce the Composite F1 Mean. Let $C_{\mathrm{comp}}$ denote the index set of all composite fault categories. The Composite F1 Mean is the arithmetic mean of the F1-scores exclusively for these composite categories:

(25) $$\mathrm{Composite}\;F1\;\mathrm{Mean}=\frac{1}{\left|C_{comp}\right|}\sum_{c\in C_{comp}}F1_{c}$$

where $\left|C_{\mathrm{comp}}\right|$ represents the total number of composite fault combinations. Furthermore, $F1_{123}$ isolates the F1-score for the most severe extreme scenario where all three basic faults overlap simultaneously, corresponding precisely to $F1_{c}$ where $c\in C_{comp}$. These metrics are critical indicators of feature decoupling capability.

### 3.2. Effect of Time-Series Image Encodings

To evaluate the influence of different time-series image encodings on compound gear fault diagnosis, five RGB-format input representations were compared on the balanced dataset: GASF, GADF, MTF, GASF-GADF, and GASF-GADF-MTF representations. For the GASF, GADF, and MTF representations, the corresponding encoding matrix was duplicated across the three RGB channels to maintain the same network input dimension. For the GASF-GADF representation, GASF and GADF were assigned to the first and second channels, respectively, while the third channel was set to zero. For the GASF-GADF-MTF representation, GASF, GADF, and MTF were assigned to the three channels, respectively. All representations were generated using the same data partitioning, window selection, dimensionality reduction, normalization, image quality screening, and class balancing procedures.

As shown in Table 5, the MTF representation produced the weakest diagnostic performance, indicating that state transition information alone was insufficient to characterize the fault-related vibration patterns. Compared with the MTF representation, both GASF and GADF inputs achieved markedly better performance, suggesting that angular correlation and angular difference information are more effective for representing the time-dependent characteristics of FBG vibration responses.

The combined representations further improved the diagnostic performance. Compared to the GASF representation, GASF-GADF increased the Exact Match Accuracy from 0.9677 to 0.9907 and improved F1_123_ from 0.8833 to 0.9722, demonstrating the complementary effect of the two Gramian angular fields. After further incorporating MTF as the third channel, GASF-GADF-MTF achieved the best overall performance, with the highest Exact Match Accuracy, Macro-F1, Composite F1 Mean, and F1_123_. In particular, F1_123_ reached 0.9901, indicating that the three-channel representation was more effective in identifying the triple compound fault class. Therefore, GASF-GADF-MTF was selected as the input representation for the subsequent experiments.

### 3.3. Comparison of Long-Tailed Distributions and Loss Functions

After determining the GASF-GADF-MTF three-channel input representation, different loss functions were further compared under long-tailed distributions. Specifically, BCE, Weighted BCE, and Focal Loss were used to train the model on the LT10, LT20, and LT50 training sets, and all models were evaluated on the same test set.

For the multi-label classification task, let $y_{ij}\in \{0,1\}$ denote the ground-truth label of the *j*-th label for the *i*-th sample, and let $p_{ij}\in [0,1]$ denote the corresponding predicted probability. Let *N* and *L* denote the number of samples and the number of labels, respectively. The standard BCE loss is defined as

(26) $$L_{\mathrm{BCE}}=-\frac{1}{NL}\sum_{i=1}^{N}\sum_{j=1}^{L}\left[y_{ij}\log(p_{ij})+(1-y_{ij})\log(1-p_{ij})\right]$$

Weighted BCE [15] introduces a positive-sample weight into BCE, which is formulated as

(27) $$L_{\mathrm{WBCE}}=-\frac{1}{NL}\sum_{i=1}^{N}\sum_{j=1}^{L}\left[w_{j}y_{ij}\log(p_{ij})+(1-y_{ij})\log(1-p_{ij})\right]$$

where *w_j_* denotes the positive-sample weight for the (*j*)-th label, which can be determined by the ratio between the numbers of negative and positive samples for that label:

(28) $$w_{j}=\frac{N_{j}^{-}}{N_{j}^{+}}$$

where $N_{j}^{+}$ and $N_{j}^{-}$ represent the numbers of positive and negative samples for the *j*-th label, respectively. The definition of Focal Loss has been given in the Methods section and is not repeated here. The diagnostic results obtained using the three loss functions under different long-tailed distributions are shown in Table 6.

As shown in Table 6, the three loss functions exhibited different levels of robustness under different degrees of long-tailed imbalance. Overall, Focal Loss achieved the best performance across all three long-tailed settings. Under the LT10 setting, Focal Loss obtained the highest Exact Match Accuracy of 0.9813, the lowest Hamming Loss of 0.0071, the highest Macro-F1 of 0.9928, the highest Composite F1 Mean of 0.9698, and the highest F1_123_ of 0.9561. Under the LT20 setting, Focal Loss also outperformed BCE and Weighted BCE across all evaluation metrics, achieving an Exact Match Accuracy of 0.9822 and an F1_123_ of 0.9381. These results indicate that Focal Loss can provide more stable optimization for multi-label compound fault diagnosis as the degree of class imbalance increases.

Under the severe LT50 setting, the advantage of Focal Loss became more evident. Compared to BCE and Weighted BCE, Focal Loss achieved the highest Exact Match Accuracy of 0.9739, the lowest Hamming Loss of 0.0092, the highest Macro-F1 of 0.9907, the highest Composite F1 Mean of 0.9613, and the highest F1_123_ of 0.9469. In particular, the F1_123_ value was markedly higher than those obtained by BCE and Weighted BCE, indicating that Focal Loss is more effective in recognizing the extreme minority triple compound fault class.

Considering the consistent superiority of Focal Loss across LT10, LT20, and LT50, and especially its stronger performance on the triple compound fault class under the severe LT50 condition, the subsequent attention module comparison was conducted using the LT50 dataset with Focal Loss.

### 3.4. Comparison of Attention Modules Under the LT50 Setting

To evaluate the influence of attention mechanisms on compound gear fault diagnosis under severe long-tailed conditions, four attention configurations were compared on the LT50 dataset with Focal Loss: none, SE [18], ECA [19], and CBAM [20]. All models used the same GASF-GADF-MTF three-channel input representation, identical data partitioning, and training protocol.

As shown in Table 7, the SE module achieved the best performance among all attention configurations, improving both overall multi-label metrics and tail triple compound fault recognition with an F1_123_ of 0.9469. ECA performed comparably on some metrics but showed lower tail-class F1. CBAM exhibited the weakest overall performance. Based on these results, SE was selected as the attention mechanism for the final backbone evaluation in the subsequent comparison with other network architectures.

### 3.5. Comparison of Different Networks Under the LT50 Setting

After selecting the GASF-GADF-MTF input representation, additional comparative experiments were conducted under the severe LT50 setting to evaluate the proposed framework against both generic backbone networks and representative long-tailed or multi-label imbalance learning strategies. The compared methods included the proposed framework, EfficientNet, ViT, ResNet18-SE with PaCo-style Prototype Contrastive Learning, and ResNet18 [21]-SE with Distribution-Balanced Loss. For fair comparison, all methods were trained and tested on the same LT50 dataset using identical data partitions, input representations, and evaluation metrics.

As indicated in Table 8, the proposed framework achieved the best overall performance under the severe LT50 long-tailed setting, with the highest Exact Match Accuracy, Macro-F1, Composite F1 Mean, and F1_123_, as well as the lowest Hamming Loss. Compared to EfficientNet and ViT, the proposed framework showed a clear advantage in compound fault recognition, especially for the extreme tail class 123. The F1_123_ value of the proposed framework reached 0.9469, whereas EfficientNet and ViT obtained only 0.2092 and 0.3987, respectively, indicating that generic backbone networks were less stable for tail compound fault recognition under severe long-tailed distributions.

Compared to the representative long-tailed and multi-label imbalance learning baselines, the PaCo-style Prototype Contrastive Learning and Distribution-Balanced Loss methods achieved competitive performance, with Exact Match Accuracy values of 0.9675 and 0.9622, respectively. However, their F1_123_ values were 0.8858 and 0.8756, respectively, which were still lower than that of the proposed framework. These results indicate that the proposed framework provides more stable feature learning and tail-class recognition for long-tailed multi-label compound gear fault diagnosis.

### 3.6. Confusion Matrix and Feature Visualization

**Figure 9:**
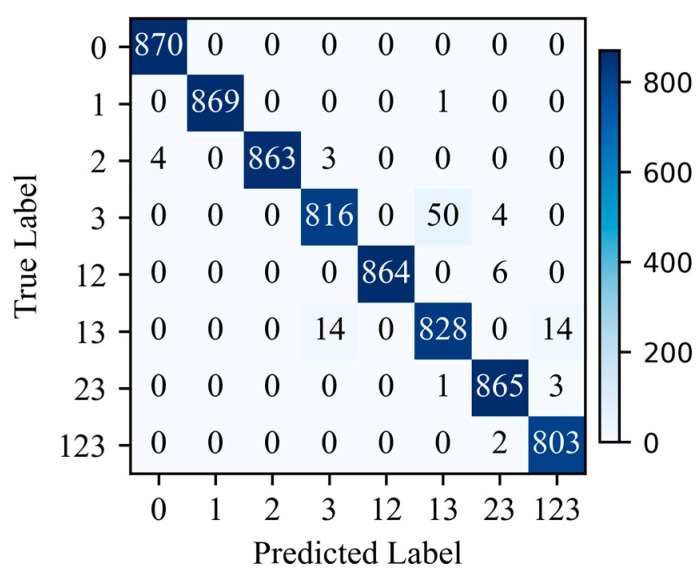
Confusion matrix of the proposed ResNet18-SE model on the balanced test set under the LT50 long-tailed setting.

To further evaluate the classification performance of the proposed ResNet18-SE model under the extreme long-tailed LT50 distribution, a confusion matrix was generated on the test set, as shown in Figure 9. Overall, the vast majority of the test samples are located along the main diagonal, indicating high true-positive rates across all categories. For the extreme minority tail class, namely the triple compound fault ‘123’, the model correctly classified 803 out of 870 samples. Regarding misclassifications, the most noticeable off-diagonal elements occurred between the single fault ‘3’ and the dual compound fault ‘13’. Specifically, 50 samples of fault ‘3’ were mispredicted as ‘13’, and 14 samples of ‘13’ were mispredicted as ‘3’. Additionally, minor misclassifications are observed between class ‘3’ and class ‘123’.

**Figure 10:**
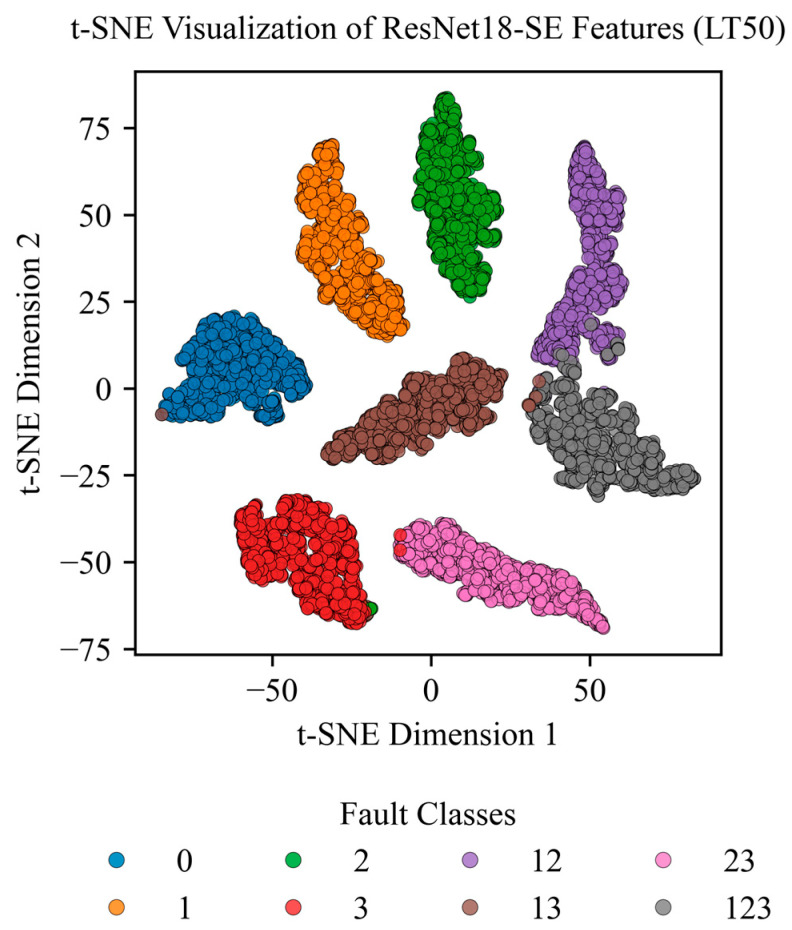
t-SNE visualization of the deep features extracted by the proposed ResNet18-SE model under the LT50 setting.

In addition to the confusion matrix, a t-SNE dimensionality reduction algorithm was utilized to project the high-dimensional features extracted prior to the final fully connected layer into a two-dimensional space to qualitatively observe the feature representation capability of the model. Figure 10 illustrates the visualization results on the LT50 test set. As observed, the extracted features are well-clustered into eight distinct groups, visually demonstrating excellent intra-class compactness and inter-class separability. The extreme minority class ‘123’ depicted by gray scatter points forms an independent cluster, and this specific cluster does not visually overlap with majority classes such as the normal state ‘0’ or single faults. Topologically, the cluster of the triple fault ‘123’ is spatially distributed in close proximity to the cluster of the dual compound fault ‘12’ in purple and the dual compound fault ‘13’ in brown.

### 3.7. Computational Complexity Analysis

To further evaluate the computational cost of the proposed model, the model parameters, computational complexity, and inference latency were measured using an input size of 3 × 224 × 224. The ResNet18-SE model contains 11.30 M trainable parameters and requires approximately 1.81 GMACs, corresponding to 3.63 GFLOPs when one multiply-add operation is counted as two floating-point operations. With a batch size of 1, the average inference latency was 7.654 ms per sample on the NVIDIA GeForce RTX 5060 Laptop GPU, measured after 50 warm-up runs and 200 repeated forward passes. These results indicate that the model can perform real-time inference on the workstation GPU used in this study.

## 4. Discussion

The results demonstrate that the multi-label formulation is effective for representing compound gear faults in wind turbine gearboxes. By treating missing tooth, pitting, and tooth breakage as independent fault components, the model can identify the coexistence of multiple faults rather than forcing each compound condition into a single mutually exclusive class. This is particularly useful for interpreting compound fault samples, where different fault components may contribute simultaneously to the measured FBG vibration response.

The superior performance of the GASF-GADF-MTF representation suggests that compound faults are reflected not only in signal amplitude variations but also in temporal correlations, local dynamic disturbances, and state transition patterns of the FBG response. Compared to single-channel encodings, the fused representation achieved better recognition performance, especially for compound fault classes, indicating that these complementary image features improve the separability of superimposed fault patterns.

The comparison of loss functions confirms that long-tailed data distribution significantly affects compound fault diagnosis. Focal Loss provided more stable performance than BCE and Weighted BCE across LT10, LT20, and LT50, particularly for the triple compound fault class. This result indicates that reducing the dominance of easily classified head-class samples is important when tail classes contain limited samples and multiple superimposed fault components.

The attention module comparison indicates that SE attention is better matched with the GASF-GADF-MTF representation than ECA and CBAM under the current long-tailed setting. Since the three channels describe the same FBG signal from different perspectives, their contributions may vary across fault types and fault combinations. The results suggest that channel-wise feature recalibration is sufficient and stable for this task, whereas more complex attention operations may not provide additional benefits when tail-class samples are limited.

The backbone comparison indicates that ResNet18-SE is more suitable than EfficientNet and ViT under the current data conditions. ViT may require larger-scale training data to learn stable global attention patterns, whereas the extreme tail classes in this study contain limited samples. EfficientNet showed lower robustness when fault-related texture differences were subtle and the class distribution was highly imbalanced. These results suggest that local convolution, residual learning, and channel attention provide a more stable feature extraction structure for limited and imbalanced diagnostic data.

The confusion matrix and t-SNE visualization further reveal the diagnostic characteristics of the proposed model. Most samples are correctly classified, while the main misclassification occurs between class 3 and class 13. As shown in Figure 6, the triple compound fault signal exhibits stronger amplitude fluctuations, more obvious impact components, and increased non-stationary characteristics compared to the single missing-tooth condition. These strong impact components may overlap with the missing-tooth-related periodic disturbance in the time-domain response, making visual separation more difficult. This provides an intuitive explanation for the partial masking phenomenon between tooth-breakage-dominated and missing-tooth-related fault patterns. This suggests that, in compound fault diagnosis, the model must not only identify whether each basic fault component is present, but also learn the coupled and partially overlapping vibration patterns produced by different fault components.

From an engineering perspective, the proposed method provides a feasible diagnostic framework for FBG-based wind turbine gearbox condition monitoring. FBG sensors offer electromagnetic immunity, multiplexing capability, low transmission loss, and suitability for long-distance monitoring. Combined with multi-channel time-series imaging and multi-label learning, the proposed framework can directly output the presence of each basic fault component, which is beneficial for fault localization, maintenance decision-making, and operation prioritization.

Nevertheless, several limitations remain. First, the experimental data were collected under a fixed-speed condition, and the generalization ability of the model under variable speed, variable load, and field operating environments requires further validation. Second, the long-tailed datasets were constructed by downsampling the balanced dataset, which cannot fully reproduce the uncertainty and noise characteristics of real long-term monitoring data. Third, the current study focuses on fault category identification and has not yet been extended to fault severity assessment or remaining useful life prediction. In addition, practical online monitoring requires both diagnostic accuracy and computational efficiency. Recent SHM studies, such as TinyLSN, have shown the importance of lightweight network design for IoT edge deployment [26]. Moreover, this study uses a single FBG sensor and does not model spatial relationships among multiple sensing locations. Adaptive graph learning methods provide a useful reference for future multi-sensor collaborative diagnosis by capturing spatial–topological relationships among sensors [27]. Future work will focus on variable-condition validation, lightweight deployment, multi-sensor graph-based modeling, and quantitative interpretability analysis to improve the applicability of the proposed framework in practical wind turbine gearbox monitoring.

## 5. Conclusions

This study proposed a long-tailed multi-label diagnostic framework for compound gear faults in wind turbine gearboxes based on FBG vibration signals and GASF-GADF-MTF multi-channel imaging. The results demonstrate that the proposed framework can effectively identify healthy, single-fault, double compound fault, and triple compound fault conditions. By encoding missing tooth, pitting, and tooth breakage as independent labels, the model can directly recognize the basic fault components contained in compound fault states.

The GASF-GADF-MTF representation effectively transforms one-dimensional FBG vibration responses into discriminative two-dimensional image patterns. Compared to single-channel and two-channel image encodings, the fused three-channel representation achieved the best diagnostic performance on the Balanced dataset, with an Exact Match Accuracy of 0.9950, a Hamming Loss of 0.0020, a Macro-F1 of 0.9980, a Composite F1 Mean of 0.9929, and an F1-score of 0.9901 for the triple compound fault class.

Under the severe LT50 long-tailed setting, the ResNet18-SE model trained with Focal Loss achieved an Exact Match Accuracy of 0.9739, a Hamming Loss of 0.0092, a Macro-F1 of 0.9907, a Composite F1 Mean of 0.9613, and an F1-score of 0.9469 for the triple compound fault class. These results indicate that the proposed FBG-based multi-channel imaging framework is effective for the long-tailed multi-label diagnosis of compound gear faults. However, this study was conducted under fixed-speed laboratory conditions, and future work will further validate the method under variable speed, variable load, and field operating environments.

## Figures and Tables

**Table 1:** Main parameters of the hinge-type FBG accelerometer.

Sensor Type	Effective Frequency Range	Sensitivity	Linearity
Hinge-type FBG accelerometer	5–1000 Hz	31.49 pm/g	>0.999

**Table 2:** Main parameters of the FBG interrogator.

Model	Number of Channels	Sampling Rate	Wavelength Range	Wavelength Resolution
YOFC-FBG-8-100	8	1 k	1525–1605 nm	0.1 pm

**Table 3:** Definition of operating conditions and multi-label fault vectors.

Class Label	Basic Fault Components	Fault Vector (F_MT_, F_P_, F_BT_)	Fault Category
0	Healthy	(0, 0, 0)	Normal
1	High-speed shaft gear missing tooth	(1, 0, 0)	Single fault
2	First-stage planetary gear pitting	(0, 1, 0)	Single fault
3	Second-stage planetary gear partial tooth breakage	(0, 0, 1)	Single fault
12	High-speed shaft gear missing tooth + first-stage planetary gear pitting	(1, 1, 0)	Compound fault
13	High-speed shaft gear missing tooth + second-stage planetary gear partial tooth breakage	(1, 0, 1)	Compound fault
23	First-stage planetary gear pitting + second-stage planetary gear partial tooth breakage	(0, 1, 1)	Compound fault
123	High-speed shaft gear missing tooth + first-stage planetary gear pitting + second-stage planetary gear partial tooth breakage	(1, 1, 1)	Triple compound fault

**Table 4:** Training sample distributions of the balanced and long-tailed datasets.

Dataset	Healthy	Single Fault	Double Compound Fault	Triple Compound Fault
Balanced	4060	4060	4060	4060
LT10	4060	1884	874	406
LT20	4060	1495	550	203
LT50	4050	1100	299	81

**Table 5:** Diagnostic performance of different time-series image representations on the Balanced dataset.

Input Representation	Exact Match Accuracy	Hamming Loss	Macro-F1	Composite F1 Mean	F1_123_
GASF	0.9677	0.0114	0.9885	0.9409	0.8833
GADF	0.9822	0.0060	0.9939	0.9731	0.9500
MTF	0.8194	0.0803	0.9179	0.6744	0.4435
GASF-GADF	0.9907	0.0033	0.9967	0.9844	0.9722
GASF-GADF-MTF	0.9950	0.0020	0.9980	0.9929	0.9901

**Table 6:** Diagnostic performance under different long-tailed distributions and loss functions.

Dataset	Loss Function	Exact Match Accuracy	Hamming Loss	Macro-F1	Composite F1 Mean	F1_123_
LT10	BCE	0.9726	0.0095	0.9905	0.9621	0.9513
LT10	Weighted BCE	0.9795	0.0077	0.9923	0.9686	0.9550
LT10	Focal Loss	0.9813	0.0071	0.9928	0.9698	0.9561
LT20	BCE	0.9779	0.0077	0.9922	0.9616	0.9291
LT20	Weighted BCE	0.9776	0.0079	0.9921	0.9605	0.9254
LT20	Focal Loss	0.9822	0.0062	0.9937	0.9677	0.9381
LT50	BCE	0.9576	0.0149	0.9850	0.9259	0.8592
LT50	Weighted BCE	0.9560	0.0150	0.9848	0.9228	0.8523
LT50	Focal Loss	0.9739	0.0092	0.9907	0.9613	0.9469

**Table 7:** Diagnostic performance of different attention modules under the LT50 dataset with Focal Loss.

Attention Module	Exact Match Accuracy	Hamming Loss	Macro-F1	Composite F1 Mean	F1_123_
None	0.9550	0.0159	0.9837	0.9166	0.8317
SE	0.9739	0.0092	0.9907	0.9613	0.9469
ECA	0.9466	0.0183	0.9813	0.9133	0.8473
CBAM	0.9444	0.0211	0.9786	0.9030	0.8269

**Table 8:** Diagnostic performance comparison with generic backbone networks and representative long-tailed or multi-label imbalance learning strategies under the LT50 setting.

Model	Exact Match Accuracy	Hamming Loss	Macro-F1	Composite F1 Mean	F1_123_
Proposed framework	0.9739	0.0092	0.9907	0.9613	0.9469
EfficientNet [22]	0.8193	0.0676	0.9263	0.6323	0.2092
ViT [23]	0.8063	0.0731	0.9217	0.6581	0.3987
ResNet18-SE + PaCo-style Prototype Contrastive Learning [24]	0.9675	0.0115	0.9884	0.9410	0.8858
ResNet18-SE + Distribution-Balanced Loss [25]	0.9622	0.0128	0.9871	0.9332	0.8756

## Data Availability

The original contributions presented in this study are included in the article. Further inquiries can be directed to the corresponding author.

## Abbreviations

The following abbreviations are used in this manuscript:

FBG	Fiber Bragg Grating
GASF	Gramian Angular Summation Field
GADF	Gramian Angular Difference Field
MTF	Markov Transition Field
CNN	Convolutional Neural Network
SE	Squeeze-and-Excitation
ECA	Efficient Channel Attention
CBAM	Convolutional Block Attention Module
BCE	Binary Cross-Entropy
LT	Long-Tailed
t-SNE	t-distributed Stochastic Neighbor Embedding
